# Foodservice Models and Nutrition Practices in Spinal Facilities: Insights and Opportunities for Improvement From a Pilot Multinational Survey

**DOI:** 10.1111/jhn.70115

**Published:** 2025-09-01

**Authors:** Isabella B. Morgan, Yilin Wang, Murray Fisher, Peter F. Collins, Priya Iyer

**Affiliations:** ^1^ Nutrition and Dietetics, Susan Wakil School of Nursing and Midwifery, Faculty of Medicine and Health University of Sydney Sydney New South Wales Australia; ^2^ Charles Perkins Centre University of Sydney Sydney New South Wales Australia; ^3^ Susan Wakil School of Nursing and Midwifery, Faculty of Medicine and Health University of Sydney Sydney New South Wales Australia; ^4^ School of Biomedical, Nutritional and Sports Sciences, Faculty of Medical Sciences Newcastle University Newcastle UK; ^5^ Digital Transformation and Research Gold Coast Hospital and Health Service Southport Queensland Australia

**Keywords:** dining models, foodservice, food systems, hospital menu, nutrition practices, nutrition standards, spinal cord injury

## Abstract

**Introduction:**

Individuals with spinal cord injury (SCI) often experience prolonged hospitalisations and are almost entirely reliant on the foodservices provided by these facilities for their nutrition and hydration needs. However, the impact of foodservice systems and dining models on patients with SCI remains poorly understood. This study aimed to investigate foodservice systems and practices employed across spinal inpatient facilities.

**Methods:**

A descriptive, cross‐sectional online survey was completed anonymously by purposively recruited dietitians employed in spinal inpatient facilities worldwide. The primary measures included aspects of foodservice systems, dining models and related nutrition practices, such as the nutritional targets of the menu, and other clinical foodservice‐related information. Quantitative data were analysed using descriptive statistics, including percentages and frequencies, while qualitative free‐text responses were evaluated using conventional content analysis.

**Results:**

Sixteen dietitians from five countries participated in the survey, with the majority representing rehabilitation facilities (10/16; 63%). These facilities reported in‐house, cook‐fresh food production systems and café‐style dining models (6/10; 60%). A daily energy target for the menu, ranging from 1500 to 2700 kcal/day, was reported by 12/16 facilities (75%). A daily protein target of 80–110 g/day was reported by 10/16 facilities (63%), while only three facilities (19%) reported having a daily variable saturated fat target.

**Conclusion:**

This novel study provides valuable insights into foodservices within spinal inpatient facilities, highlighting variability in the nutrient goals of menus across different sites. Particularly, the limited focus on saturated fat, despite the elevated cardiovascular risk in this population needs consideration. Future research should explore these issues in a larger, diverse sample and work toward standardising tangible nutrition practices to improve the quality of care and nutritional outcomes.

## Practitioner Points

- Foodservice systems and dining models are the core of nutrition management in hospitals and should be considered in both research and practice.
- Standardisation of nutrient targets of the menus as a minimum is necessary for consistent care and outcomes.
- Emphasis on crucial nutrients, such as saturated fat, is needed to ensure a health‐promoting menu for patients in spinal facilities.

## Graphical abstract

A visual snapshot of the study

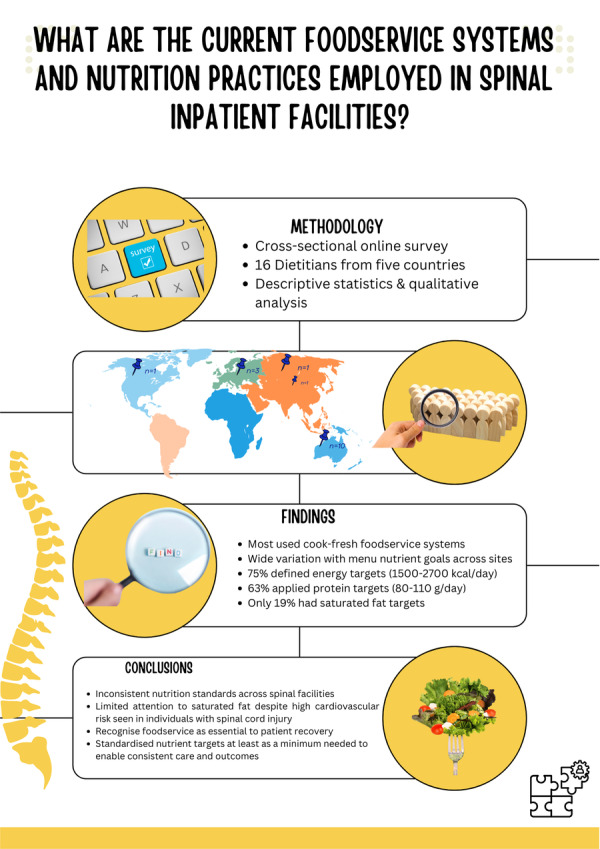

## 1 Introduction

Spinal cord injury (SCI) is associated with a range of metabolic changes [1], which have been associated with increased risks in obesity [2], Type 2 Diabetes [3] and cardiovascular disease (CVD) [4]. Alterations in body composition following SCI, characterised by a reduction in lean body mass and an increase in adipose tissue, are key contributors to these metabolic sequelae [1]. The resultant changes to energy metabolism [5], combined with factors such as reduced physical activity due to functional limitations [6], underscore the need for an energy‐balanced and nutritionally adequate diet to help counter these effects [7].

Obesity has a greater prevalence in people with SCI compared to the general population [8], with reports indicating that rates are up to four times higher among individuals with disabilities, including those with SCI [9]. Evidence shows that many individuals with SCI have excessive energy intake relative to their energy expenditure [5, 10], and a moderate 15%–20% calorie deficit is suggested to maintain ideal body composition and prevent cardiometabolic disturbances following SCI [7]. While the literature does not provide a clear timeline for the duration of the energy deficit, this approach may be particularly beneficial during rehabilitation, as energy expenditure tends to remain consistently low in this population [11]. However, with growing recognition of the prevalence and effects of sarcopenic obesity in people with SCI [12], careful consideration of both calorie restriction and muscle preservation is essential.

Furthermore, consumption of diets high in energy, saturated fat and low in essential food groups, namely vegetables and wholegrains, has been reported in this population [13, 14]. Excessive energy intake and poor dietary patterns seen in this population [10, 13, 14] adversely affect individual recovery trajectories and increase strain on healthcare systems [15]. Reduced oral intake lacking key nutrients can lead to malnutrition [16], which in turn may significantly affect health outcomes and contribute to increased hospitalisations and longer length of stay (LOS) [17]. LOS indirectly shapes dietary behaviours by determining whether food choices are made within a controlled institutional environment or in the more variable home setting [13]. These differences can significantly impact nutrition outcomes, highlighting the importance of optimal nutrition in preventing and treating both malnutrition and obesity.

The association between SCI and prolonged LOS is attributed to medical complications and reduced functional improvement [17]. In Australian rehabilitation facilities, the LOS for individuals with SCI increased from 76.6 days in 2022 to 89.7 days in 2024 [18]. However, LOS varies significantly across the globe [19]. Long‐stay admissions present unique challenges to rehabilitation facilities, as residents are typically reliant on the foodservice for all their hydration and nutrition needs. Therefore, the responsiveness of hospital foodservice systems and dining models is critical to delivering optimal nutrition care. Despite this importance, little is known about the dining models and foodservice operations in healthcare settings that care for people with SCI. A recent scoping review has highlighted this gap in the literature [20]. In response, this study aimed to explore current foodservice systems and related nutrition practices in spinal inpatient facilities.

## 2 Materials and Methods

A descriptive, cross‐sectional online survey methodology was used for this pilot study, and appropriate ethical approval was obtained from the University of Sydney Human Research Ethics Committee (2024/HE000150). The study was conducted in Sydney, New South Wales, Australia. Recruitment used a combination of purposeful and snowball sampling strategies [21], targeting dietitians proficient in English and employed within spinal inpatient facilities globally via international professional networks. Given that spinal dietetics is a niche area with a membership of approximately 20 dietitians within the international spinal dietitians' network, an 80% response rate was determined for the study. Participation was voluntary, and participants were informed that no reimbursement would be provided. Participants could withdraw from the study at any point before submitting the survey; however, withdrawal was not possible after submission and data analysis due to the anonymous nature of the survey.

The online survey was hosted on the secure Research Electronic Data Capture (REDCap) platform [22, 23] and remained open for 3 weeks. The questionnaire (Supporting Information S1) was devised by the primary investigator (PI) with subject matter expertise and experience in real‐world clinical settings, with questions based on local practice standards [24, 25] and relevant literature. The questionnaire consisted of four sections. The first two sections gathered basic demographic information about the participant and the facility (e.g., location, type of hospital, number of spinal rehab beds, etc.). The third section focussed on foodservice systems, while the final section addressed nutrition practices related to foodservices and dietary assessment. The questionnaire, which was enabled as an online survey, was tested for face and content validity by two student dietitians external to the research team. Additionally, content validity was evaluated through a pilot test conducted with two experienced dietitians possessing subject matter expertise (Supporting Information S2). Based on the feedback received, several questions were refined for improved clarity and relevance using an iterative approach by the research team members (P. I., M. F. and P. F. C.). For example, when ‘Other’ was selected as a response option, a follow‐up prompt was added to encourage elaboration. Supporting Information S2a includes a summary of the content validity results, along with specific feedback and corresponding revisions. Data were categorised by facility type to compare foodservice systems and nutrition practices. All data were analysed in Statistical Package for the Social Sciences (IBM SPSS version 29) and reported descriptively using frequencies and percentages. Textual comments were managed using Microsoft Excel (Microsoft 365) and analysed using a simple conventional content analysis, enabling the identification of relevant themes [26, 27]. All participants' quotes were downloaded into Microsoft Excel, and each participant's quote was considered as a meaning unit, which was read and re‐read to identify codes from which key themes or broader categories were directly derived. This method was opted as the most suitable approach as it enables direct coding from the texts to drive analysis of participants' perspectives [26, 27].

## 3 Results

### 3.1 Characteristics of Participants

A total of 16 dietitians participated in the multinational survey, representing an 80% response rate, with almost all providing complete responses (*n* = 15/16; 94%). These dietitians came from various settings, including acute care (*n* = 3/16; 19%), rehabilitation (*n* = 10/16; 63%), combination (*n* = 1/16; 6%) and unknown setting (*n* = 2/16; 13%). Many participants were from Australia (*n* = 10/16; 63%), followed by the United Kingdom (*n* = 2/16; 13%), Norway (*n* = 1/16; 6%), Canada (*n* = 1/16; 6%) and Nepal (*n* = 1/16; 6%); one location was unknown, representing diverse geographical locations. Rehabilitation settings accounted for a significant portion (*n* = 10/16; 63%), with most facilities being ≤ 30 beds (*n* = 9/16; 56%) in capacity. The LOS across all participating sites ranged from 30 to 365 days (Table 1).

**Table 1:** Characteristics of participants.

Characteristics	*n* (%)
Years of professional experience	
< 1–10 years	9 (60)
> 10 years	6 (40)
Facility location	
Metropolitan	12 (80)
Rural	3 (20)
Facility type	
Acute	3 (20)
Rehabilitation	10 (67)
Other^a^	2 (13)
Bed numbers	
Acute	
≤ 30 beds	2 (13)
> 30 beds	1 (7)
Rehabilitation	
≤ 30 beds	6 (40)
> 30 beds	4 (27)
Unknown	
> 30 beds	1 (7)
Combination	
≤ 30 beds	1 (7)
Length of stay (days)	
Acute	
30–200 days	3 (20)
201–400 days	—
Rehabilitation	
30–200 days	7 (47)
201–400 days	1 (7)
Unknown	2 (13)
Other^a^	
30–200 days	2 (7)
201–400 days	

*Note:* Data are presented as *n* (%). Percentages are rounded.

^a^ Other = co‐located facility (*n* = 1) and unspecified setting (*n* = 1).

### 3.2 Foodservice Systems and Processes

Table 2 highlights foodservice systems and dining models, noting the key differences in foodservice systems between acute and rehabilitation facilities. Rehabilitation facilities primarily cooked meals onsite (*n* = 6/10; 60%) compared to acute facilities (*n* = 1/3; 33%). Tray service was the common distribution system in both settings, but rehabilitation facilities were more likely to use a mix of service delivery models (*n* = 2 vs. acute *n* = 0). A café‐style dining model, which generally encompass service approaches where meals are provided in a hospital cafeteria accessible to patients, visitors and staff [28], as well as menu designs and service formats that resemble those found in restaurants or bistro [29] were used in many rehabilitation facilities (*n* = 7/10; 70%) compared to acute facilities (*n* = 1/3; 33%). However, only a few facilities reported employing a room service model (*n* = 2/15; 13%). Traditional three main meals and midmeals were common practice in all acute facilities, four rehabilitation centres (40%), and one co‐located facility. Both types of facilities offered a variety of mid‐meals and used biodegradable materials in foodservice, such as reusable crockery and cutlery (Supporting Information S3).

**Table 2:** Food systems and dining models.

Food and dining systems	All facilities (*n* = 15)	Acute (*n* = 3)	Rehabilitation (*n* = 10)	Others^a^ (*n* = 2)
Production and distribution systems				
Cooked onsite	8 (53)	1 (33)	6 (60)	1 (50)
Cooked offsite	5 (33)	2 (67)	2 (20)	1 (50)
Purchased in	1 (7)		1 (10)	
Combination	1 (7)		1 (10)	
Service delivery				
Tray service	10 (67)	3 (100)	6 (60)	1 (50)
Self or table service	3 (20)		2 (20)	1 (7)
Combination	2 (13)		2 (20)	
Dining environment				
Communal dining	6 (40)		5 (50)	1 (50)
Bedside dining	4 (27)	2 (67)	2 (20)	
Combination	6 (40)	1 (33)	3 (30)	1 (50)
Dining models				
Cafe style	9 (60)	1 (33)	7 (70)	1 (50)
Room service	2 (13)	1 (33)		1 (50)
Traditional tray service	4 (27)	1 (33)	3 (30)	
Midmeal/s				
Yes	10 (67)	3 (100)	5 (50)	2 (100)
No			5 (50)	
Menu				
CBORD	8 (53)	1 (33)	6 (60)	1 (50)
Chefmax/delegate/label logic	5 (33)	1 (33)	4 (40)	
None	2 (13)	1 (33)		1 (50)
Menu format				
Spoken/digital menu	2 (13)	1 (33)	1 (10)	
Paper‐based menu	6 (40)	1 (33)	3 (30)	2 (100)
Combination		1 (33)	6 (60)	
Menu cycle				
1–2 weeks	9 (60)	2 (67)	5 (50)	2 (100)
4–6 weeks	6 (40)	1 (33)	5 (50)	
Seasonal menu				
Yes	2 (13)		1 (10)	1 (50)
No	13 (87)	3 (100)	9 (90)	1 (50)
Menu frequency				
Daily	12 (80)	3 (100)	7 (70)	2 (100)
Weekly	2 (13)		2 (20)	
Virtual display	1 (7)		1 (10)	
Menu design				
Nutrition/Menu standards	12 (80)	3 (100)	8 (80)	1 (50)
National dietary guidelines	1 (7)		1 (10)	
Combination	1 (7)		1 (10)	
Nil standards	1 (7)			1 (50)
Meal patterns				
3× main meals + 3× midmeals	8 (53)	3 (100)	4 (40)	1 (50)
3× main meals only	2 (13)		2 (20)	
Other^b^			4 (40)	1 (50)
Therapeutic diets				
< 20	5 (33)	1 (33)	3 (30)	1 (50)
20–70	8 (53)	1 (33)	7 (70)	
> 70	2 (13)	1 (33)		1 (50)
Texture‐modified diets				
< 5	2 (13)		1 (10)	1 (50)
5–10	11 (73)	3 (100)	8 (80)	
> 10	2 (13)		1 (10)	1 (50)

*Note:* Data are presented as *n* (%). Percentages are rounded.

^a^ 1× co‐located facility and 1× unknown facility type.

^b^ Four main meals (*n* = 1), 3× main meals and self‐served snacks (*n* = 3), combination (*n* = 1).

Menu management systems were used in all rehabilitation facilities (*n* = 10/10; 100% vs. acute *n* = 2), with CBORD being the most common system (*n* = 6/10; 60% of rehabilitation facilities) and 53% of all facilities collectively (*n* = 8/15). Rehabilitation facilities also employed a greater variety of menu formats (spoken, digital, paper‐based), with 6/10 (60%) using a combination of these formats compared to only 1/3 (33%) in acute facilities. Overall, less than 20% (*n* = 3/16) of facilities used digital menus (rehabilitation). The menu cycle length of 1–2 weeks was most common (*n* = 9/15; 60%), although 50% (*n* = 5) of rehabilitation facilities also had a longer cycle of 4–6 weeks. Interestingly, seasonal menus were uncommon and used only in one unknown setting and one rehabilitation facility. Many facilities also offered a range of mealtime support to patients (*n* = 13/15; 87%), with 93% (*n* = 14/15) of facilities helping with eating, meal set‐up and cut‐up and provision of modified cutlery and plates. Additionally, 87% (*n* = 13/15) of facilities reported offering mealtime supervision to patients, while 47% (*n* = 7/15) offered companionship support to patients. Both types of facilities relied heavily on foodservice staff or diet aides for menu distribution (rehabilitation *n* = 7/10; 70% vs. acute *n* = 2/3; 67%), with many rehabilitation facilities having staff overseeing menu compliance with guidelines (*n* = 7/10; 70% vs. acute *n* = 1/3; 33%). Between 20 and 70 therapeutic diet menus were offered within most rehabilitation facilities (*n* = 7/10; 70%; acute *n* = 1/3; 33%), while 5–10 texture‐modified diets were available in most facilities in both settings (acute *n* = 3/3; 100%; rehabilitation *n* = 8/10; 80%). All facilities referred to nutrition or menu standards, with 80% (*n* = 12/15) reporting the use of the International Dysphagia Diet Standardisation Initiative (IDDSI) framework. Furthermore, most facilities reported employing multiple methods of foodservice evaluation (rehabilitation *n* = 7/10; 70% vs. acute *n* = 2/3; 67%) (Supporting Information S3). Patient satisfaction surveys or interviews were reported to be used in three facilities (19%), while menu assessment and plate waste audit were reported to be employed by one facility (6%).

### 3.3 Nutrition Practices

Nutrition practices related to foodservice systems are outlined in Table 3. Almost all facilities (*n* = 15/16; 94%) reported the involvement of clinical dietitians in foodservice operations. Most facilities (14/16; 88%) reported using a combination of oral intake assessment tools such as food records and diet histories. Twelve out of 16 (75%) facilities reported setting a daily energy target for their menus, with 5 facilities targeting 1801–2000 kcal/day. However, the overall range of reported energy targets varied from 1500 to 2700 kcal/day. Additionally, 10/16 (62.5%) facilities reported that their menus were guided by a daily protein target. Of these, over half (*n* = 9/16; 56%) aimed for 80–100 g a day, while one acute facility reported a target of < 80/day. In contrast, only three facilities (19%) considered a daily saturated fat target in their menu design.

**Table 3:** Nutrition practices.

Facility type	All facilities (*n* = 16)	Acute (*n* = 3)	Rehabilitation (*n* = 10)	Others^a^ (*n* = 3)
Dietetic input into foodservices				
Yes	15 (94)	3 (100)	10 (100)	2 (67)
No	1 (6)			1 (33)
Intake assessment tools^b^				
Food record/diet history/FFQ	1 (7)		1 (10)	
Combination^c^	14 (93)	3 (100)	9 (90)	2 (67)
Daily energy target				
1500–1800 kcal	3 (19)	2 (67)	1 (10)	
1801–2000 kcal	5 (31)		3 (30)	2 (67)
> 2000 kcal	4 (25)	1 (33)	3 (30)	
Not known	4 (25)		3 (30)	1 (33)
Daily protein target				
< 80 g	1 (6)	1 (33)		
80–110 g	9 (56)	2 (67)	6 (60)	1 (33)
Not known	6 (38)		4 (40)	2 (67)
Daily saturated fat target				
≤ 10% of energy	1 (6)		1 (10)	
Nil target	1 (6)		1 (10)	
Other^d^	2 (13)		2 (20)	
Not known	12 (75)	3 (100)	6 (60)	3 (100)
Menu flexibility				
Therapeutic/texture‐modified diet choices	16 (100)	3 (100)	10 (100)	3 (100)
Customised meal serves	11 (69)	2 (67)	7 (70)	2 (67)
Diet quality assessment				
Yes	9 (56)	1 (33)	6 (60)	2 (67)
No	7 (44)	2 (67)	4 (40)	1 (33)
Diet quality evaluation tools				
Australian Guide to Healthy Eating (AGHE)	2 (13)	1 (33)	1 (10)	
No tools used	2 (13)		2 (20)	
Combination^e^	3 (20)		2 (20)	1 (33)
Menu selection education				
Ward clerks/diet aides	2 (13)		2 (20)	
Dietitians	6 (38)	1 (33)	3 (30)	2 (67)
Unsure or no	1 (6)			1 (33)
Combination	7 (44)	2 (67)	5 (50)	
External food outlets				
Family/friends	2 (13)			2 (67)
Multiple sources^f^	14 (88)	3 (100)	10 (100)	1 (33)
Guidelines for external foods				
Yes	6 (38)	1 (33)	5 (50)	
No	10 (63)	2 (67)	5 (50)	3 (100)

*Note:* Data are presented as *n* (%). Percentages are rounded and may not add up to 100%.

^a^ 1× co‐located facility and 2× unknown facility type.

^b^ *n* − 15.

^c^ 24‐h recall, CBORD, Mobile intake, food record, diet history.

^d^ 30 g (*n* = 1) and < 5 g for main meals (*n* = 1).

^e^ Australian Guide to Healthy Eating, Ready reckoner, bowel management recommendations, nutrient reference values.

^f^ Vending machines, cafes/kiosks, local restaurants/takeaway shops, Uber Eats/other delivery services.

All facilities reported making efforts to accommodate patients' dietary needs; however, only 69% (*n* = 11/16) customised meal serving sizes. Various health facilities reported undertaking diet quality assessments using tools such as the Australian Guide to Healthy Eating, national dietary guidelines, and patient‐reported data as part of routine nutrition assessment of patients. Most participants (94%) reported that patients had access to food and beverages from multiple sources within the hospital, with the most common options including vending machines, cafes/kiosks, local restaurants or takeaway shops and delivery services such as Uber Eats. Most facilities (10/15; 67%) indicated that there were no specific guidelines or policies in place to regulate foods sourced from outside the hospital foodservice. While several facilities (*n* = 10/16; 63%) reported assessing the diet quality of meals, the tools used for assessment varied, and a few facilities used no specific tools.

### 3.4 Simple Content Analysis of Comments on Food Systems and Dining Models

Simple conventional content analysis [26, 27] of all additional free‐text comments (*n* = 9/16; 56%) provided in the survey (Supporting Information S4) revealed five key themes related to foodservice delivery, patient satisfaction and systemic challenges which are outlined below. The data reflected both persistent issues and emerging efforts to improve service quality (Table 4).

**Table 4:** Key themes and codes from the qualitative comments.

Themes	Codes
Foodservice system limitations	Cost, restrictive policies, lack of control by on‐site staff, minimal responsiveness from external foodservice companies and lack of staff training
Patient‐centred concerns	Patient dissatisfaction, unmet dietary needs, high wastage and long‐stay patients are overlooked
Dietitian advocacy	Ongoing efforts to improve services
System improvements	Insourcing, kitchen renovations, menu planning, consumer engagement, evidence‐based strategies
Menu and diet flexibility	Allergies, intolerances, texture diets, special orders, OWI policies

### 3.5 Foodservice System Limitations

Participants frequently described weaknesses in current foodservice systems, quoting poor management oversight, insufficient evaluation and staff training, and a lack of staffing, such as full‐time dietitians. These limitations were reported to be linked to funding constraints:

The food service management is still not so strong and needs more work out with regular monitoring and evaluation. Need of full‐time dietitian and which is still lacking due to no funding in the rehabilitation centre.

(Participant 2)

Restrictive policies, such as those discouraging the provision of externally prepared meals, were also seen to limit flexibility in person‐centred care.

Meal management procedure discourages the provision/storage of externally produced cooked meals to patients.

(Participant 8)

## 4 Patient‐Centred Concerns

Another concern reported was the widespread patient dissatisfaction with food quality, variety and service, especially among long‐stay patients. Participants reported high levels of plate waste and suboptimal nutritional intake, with dietitians expressing frustration at the lack of responsiveness from overarching foodservice providers:

Our patients are highly dissatisfied with the food quality, lack of variety and overall service … Whilst Dietetics strongly advocate for change … the overarching food service companies show little engagement. We essentially feel powerless for our patients.

(Participant 9)

This dissatisfaction was often linked to a lack of responsiveness to individual dietary needs, including allergies, modified textures and cultural or personal preferences.

## 5 Dietitian Advocacy

Dietitians consistently reported advocating for improved foodservices. However, their efforts were frequently limited by external constraints such as food safety policies and financial restrictions, which often led to inadequate solutions:

Dietitian constantly raised their concerns … Unfortunately the food service department was bound by food safety policy and cost which led to the ‘solutions’ being offered of little value to patients.

(Participant 7)

## 6 System Improvements

Despite these challenges, some facilities reported initiatives aimed at reforming their foodservice models. These included insourcing food preparation, engaging consumers in developing the menu, and planning major kitchen renovations to enable ‘cook fresh on demand’ approaches:

We are currently working on insourcing our food service to better meet our patients individual needs … This will hopefully be in place by 2030.

(Participant 3)

Our SCI rehab facility is soon to undergo a significant kitchen renovation… The goal is that it will be a combination of what consumers want and what the research tells us is beneficial from a nutrition and environment perspective.

(Participant 5)

Such initiatives reflect an emerging shift towards a more personalised, evidence‐informed foodservice models aligning with patient expectations and nutritional needs.

## 7 Menu and Diet Flexibility

While some flexibility existed, such as ‘ward extras’ lists and special diet codes, participants noted that menu design often failed to capture the complexity of dietary requirements, particularly for individual allergies, intolerances and texture modifications:

List of diets would be larger if counted all individual allergy and intolerances and included each modified texture as one diet.

(Participant 4)

## 8 Discussion

This pilot multinational survey provides preliminary insights across acute and rehabilitation settings for people with SCI, highlighting considerable variation in foodservice systems and practices even within similar care settings. Rehabilitation facilities tended to employ flexible approaches, including cooking onsite, using café‐style dining, menu management systems, and providing diverse menu formats. However, despite the involvement of clinical dietitians at all sites, generally aiming to meet dietary needs, several limitations were evident. Seasonal variety and digital menus were not common, and customised meal serve sizes were not widely available. While mealtime support was widely provided, the limited use of patient satisfaction surveys indicates a missed opportunity to evaluate and improve the patient foodservice experience routinely. The common availability of external food sources, often with no clear guidelines, also raises questions about consistency in nutritional quality and food safety. These highlight the lack of person‐centred care, which is especially important in long‐term inpatient settings and in meeting the unique nutrition needs of people with SCI [30].

This study revealed substantial variability in energy, protein and fat targets across facilities, with many not aligned to the nutritional requirements in SCI [30]. Only 75% (*n* = 12/16) of sites reported a daily energy target, 63% (*n* = 10/16) reported a protein target, and just 19% (*n* = 3/16) specified a saturated fat goal. While general nutrition standards such as those used in Australia (1912 kcal/day) [24], Canada (2000 kcal/day) [31] and the United Kingdom (1840 kcal/day) [32] inform nutrient targets in these countries, they are not SCI‐specific. Higher energy target is concerning given strong evidence that individuals with SCI have significantly reduced resting energy expenditure [33, 34] with recommended energy needs being closer to 1500 kcal/day or even lower for a given body weight, depending on the level of injury (22.7 kcal/kg/day in tetraplegia and 27.9 kcal/kg/day in paraplegia, for chronic SCI [33]). Combined with long LOS (ranging from 30 to 400 days in our study), and free access to external food sources such as vending machines, cafés, and food items from family members (reported by all sites), there is a further risk of excessive energy intake in this population. Similarly, saturated fat targets were either absent or exceeded recommended limits, with one site reporting < 13% of energy from saturated fat, which is more than twice the 5%–6% limit recommended for individuals with SCI [34, 35]. These findings point to a broader concern that while most sites (*n* = 14/15; 93%) reported using nutrition standards or dietary guidelines in menu planning, these frameworks are typically not SCI‐specific. The absence of SCI‐adapted standards may contribute to unintentional overfeeding and suboptimal macronutrient profiles, ultimately increasing the risk of obesity, CVD and type 2 diabetes conditions already disproportionately affecting this population [1, 3, 36].

Clinical dietetic practices related to foodservice systems varied across facilities. While nearly all sites (*n* = 15/16; 94%) reported dietitian involvement in foodservice operations, just over half (53%) indicated that they assess the diet quality of patients' oral intake as part of routine nutrition care. Notably, none of the facilities reported using validated diet quality measurement tools [37, 38]. This gap highlights an opportunity to improve the accuracy and consistency of nutrition assessments to guide targeted dietary interventions. Sustainability practices within foodservice systems also varied. Increased access to meals and snacks from outside sources, as reported in all facilities, raises concerns about food waste and environmental impact. Additionally, the use and disposal of single‐use plates, utensils and packaging materials contribute to environmental burdens [39, 40, 41]. In this study, six facilities reported using non‐biodegradable materials or a combination of biodegradable and non‐biodegradable options, while eight used more sustainable alternatives. A small number of sites also reported efforts to reduce environmental impact using seasonal menus [39, 40, 41, 42], although this practice remains limited; 87% of facilities did not rotate menus seasonally. Expanding sustainable practices such as biodegradable materials and seasonal menu planning could support broader health system sustainability goals.

Qualitative comments revealed significant systemic challenges impacting foodservice delivery. Dietitians cited management oversight, staffing limitations and restrictive food safety policies that inhibit person‐centred approaches. These findings echo recent studies that highlight how institutional systems often fail to accommodate individual patient needs [43, 44]. This misalignment may partly explain the increased plate waste and patient dissatisfaction reported in our survey. Emerging research also emphasises that mealtimes are more than just a method of delivering nutrition; they are integral to a patient′s rehabilitation journey, promoting autonomy, social interaction and dignity [45]. Some positive initiatives, such as insourcing, consumer co‐design of menus, and major kitchen renovations, reported in the survey, suggest an emerging shift towards more responsive and individualised foodservice models, although implementation remains uneven across sites.

The key strength of this study is that it is the first to provide novel insights into foodservice systems and nutrition practices in SCI facilities across multiple countries. With 16 responses from five countries, the study achieved its a priori target of 80% response rate from spinal dietitians within international professional networks. Furthermore, there was no duplication of facility responses, as ascertained by the unique characteristics of each representative facility in the survey. Although a snowball approach was used to increase responses beyond the established networks, the number of responses was minimal, largely due to the limited recruitment window outlined in the approved protocol. Consequently, a wider global representation was not achieved. Representation from low‐resource countries was limited, with only one response received from Nepal, which restricts the study′s ability to explore these concepts in low‐resource nations. The sample size was small, and the survey was in English, limiting responses from non‐English‐speaking nations. Additionally, the study did not focus in‐depth on sustainability as it was not the primary focus. This highlights the unmet need and opportunity for future research to examine sustainability initiatives within healthcare foodservice systems. Finally, the study gathered details on foodservices; however, the level of detail was constrained by the use of close‐ended questions in a cross‐sectional survey. While free comments provided valuable, relevant information, these could not be explored in detail due to survey design limitations. Future studies should consider employing qualitative methods such as focus groups to expand on this knowledge.

## 9 Conclusions

This pilot multinational survey provides valuable insights into foodservice and nutrition practices across spinal inpatient settings, highlighting important differences between rehabilitation and acute care. While rehabilitation facilities demonstrated better person‐centred care, offering flexible, diverse and on‐site foodservice models, persistent challenges remain. These relate to limited policy support, operational constraints and limited technological integration, all of which restrict person‐centred care. Many facilities lacked clearly defined nutrition targets, particularly for saturated fat, despite the heightened CVD risk in this population. These gaps underscore the absence of SCI‐specific nutrition guidelines, highlighting a critical area for future research and policy development.

Dietitians play a vital role in advocating for improved systems and delivering evidence‐based nutrition care, but are often hindered by funding and policy constraints. Enhancing menu responsiveness and patient meal experiences will require greater investment in staff training, personalised menu options and inclusive foodservice models. Initiatives such as insourcing and menu co‐design with consumers, and digital platforms may hold potential for addressing these complex challenges. To better meet the nutritional needs of individuals with SCI, foodservice systems must evolve. Standardising policy frameworks, expanding digital integration and actively involving patients in foodservice planning are essential steps to align care with both therapeutic goals and individual preferences for responsive, person‐centred foodservice practices.

## Author Contributions

Conceptualisation: Priya Iyer. Methodology: Priya Iyer. Software: Priya Iyer. Validation: Priya Iyer, Isabella B. Morgan and Yilin Wang. Formal analysis: Isabella B. Morgan, Yilin Wang, Priya Iyer and Murray Fisher. Investigation: Isabella B. Morgan, Yilin Wang and Priya Iyer. Resources: Priya Iyer. Data curation: Isabella B. Morgan, Yilin Wang and Priya Iyer. Writing – original draft preparation: Isabella B. Morgan and Yilin Wang. Writing – review and editing: Priya Iyer, Murray Fisher and Peter F. Collins. Visualisation: Priya Iyer, Isabella B. Morgan and Yilin Wang. Supervision: Priya Iyer, Murray Fisher and Peter F. Collins. Project administration: Priya Iyer. All authors have read and approved the final manuscript for publication.

## Ethics Statement

The study was conducted according to the guidelines of the Declaration of Helsinki and approved by the Institutional Review Board (or Ethics Committee) of UNIVERSITY OF SYDNEY (2024/HE000150 approved on 12 August 2024).

## Consent

Informed consent was obtained from all subjects involved in the study.

## Conflicts of Interest

The authors declare no conflicts of interest.

## Supporting information

Suppementary materials Food systems study final 1.

## Data Availability

The data that supports the findings of this study are presented within the main article and Supporting material of this article. All data are available within the paper.
