# Supplementary material for: Foodservice Models and Nutrition Practices in Spinal Facilities: Insights and Opportunities for Improvement From a Pilot Multinational Survey
Source: J Hum Nutr Diet. 2025 Sep 1;38(5):e70115. doi: 10.1111/jhn.70115 (PMC12402681; doi:10.1111/jhn.70115)
Supplement: Supplementary file 1 — Suppementary materials Food systems study final 1. [file JHN-38-0-s001.pdf]

Supplementary Materials

S1: Main questionnaire

S2: Excerpt of the content validity form

S2a: Summary of content validity results

S3**:** Other Aspects of Foodservice Systems and Dining Models

S4: Summary of Free-text comments

A multinational survey of foodservice systems and nutrition practices within spinal facilities

Please complete the survey below.

Thank you!

SECTION 1: YOUR DETAILS

1) I am a dietitian with either current or past experience within spinal facilities. E.g., acute or rehabilitation centres.

Yes
No

2) I have been a dietitian in the area of spinal cord injury (SCI) for

- ◯ < 1 year
- ◯ 1-5 years
- ◯ 5-10 years
- ◯ >10 years

SECTION 2: INFORMATION ABOUT YOUR FACILITY

	Acute public hospital	Acute private hospital	Rehabilitation facility (public)	Rehabilitation facility (private)	Other
3) Please select the facility associated with your current or previous workplace that will form the basis of your survey responses. Note: If worked across multiple settings, please select the one on which your survey responses will be based.	◯	◯	◯	◯	◯

4) If you had selected 'other' for the last question, please provide an explanation here.

____

5) Please provide the name of the country where the hospital/spinal facility you are working at or referring to for this survey is located.

____

6) Please indicate the geographical area of the hospital/spinal facility referred to for this survey.

- ◯ Metropolitan area
- ◯ Rural or remote area

7) Please indicate the total spinal bed numbers at the facility.

____

8) Please indicate the average length of stay (LOS) of the spinal cohort at the facility.

____

SECTION 3: FOOD SERVICE SYSTEMS & PROCESSES

9) Meals at the facility are

- ☐ cooked onsite within the hospital/facility
- ☐ cooked offsite in a production unit/kitchen
- ☐ cooked meals are purchased from contracted meal providers
- ☐ Other

---

10) If 'other' was selected for the previous question, please provide details.

____

---

11) Please identify the Food Service production system utilised at the facility.

- ◯ Conventional or traditional (meals prepared on-site)
- ◯ Commissary or centralised (meals prepared in a central production site)
- ◯ Ready-Prepared (meals prepared in advance, chill or freeze to serve later)
- ◯ Assembly-Serve (meals purchased from outside and assembled for service on-site)
- ◯ Combination

---

12) If 'combination' has been selected for the last question, please elaborate.

____

---

13) Please describe the Food Service distribution system used at the facility (e.g., centralised/decentralised; hot-plated; insulated carts etc).

____

---

14) Please select the type of meal service that best reflects the service delivery at the facility.

- ◯ Tray Service
- ◯ Self-Service (counter/buffet/cafe style etc)
- ◯ Table Service
- ◯ Other

---

15) If 'other' was selected for the last question, please provide details.

____

---

16) Please choose the meal pattern that best reflects the routine pattern at the facility. This pertains to primary meals, such as breakfast, lunch, and dinner, as well as midmeals like morning tea, afternoon tea, and supper.

- ◯ 3 x main meals and 3 x midmeals
- ◯ 3 x main meals and 2 x midmeals
- ◯ 3 x main meals only
- ◯ 2 x main meals and 2 x midmeals
- ◯ Other

---

17) If 'other' was selected for the last question, please provide details.

____

Dining Model

18) Please choose the dining environment that best represents the environment at the facility.

○ Communal dining ○ Bedside dining ○ Other

19) If 'other' was selected for the last question, please describe other.

____

20) Please choose the dining model that best describes the one used within the facility

○ Cafe-style ○ Room Service ○ 'Dining on Call' Service ○ Other

21) If 'other' was selected for the last question, please describe other.

____

	Yes	No
22) Please confirm if the dining environment identified for the facility also applies to midmeals.	○	○
23) Please confirm if the dining model identified for the facility also applies to midmeals.	○	○

24) If you've answered 'no' to the previous questions regarding the dining model and the environment for midmeals, please provide details.

____

25) Please select all items which are typically served during midmeals.

- ☐ Hot beverages (e.g., coffee, tea)
- ☐ Cold beverages (e.g., fruit juices)
- ☐ Biscuits
- ☐ Cakes and muffins
- ☐ Fresh fruits or pc fruits (tinned/packed)

26) Please choose the materials used for the plates and cutlery at the facility (select all that applies).

- ☐ Biodegradable materials
- ☐ Non-biodegradable materials
- ☐ Reuseable
- ☐ Single-use disposable

27) Please select all the methods by which drinking water is served during mealtimes at the facility.

- ☐ Plastic water bottles
- ☐ Plastic reuseable water jugs and glasses
- ☐ Reuseable glasses

28) Please identify all available mealtime support options at the facility.

- ☐ Feeding assistance
- ☐ Meal set-up
- ☐ Meal cut-up
- ☐ Modified cutlery & plate
- ☐ Companionship
- ☐ Supervision
- ☐ No mealtime support offered

Menu Management

	CBORD	CHEFMAX	DELEGATE	OTHER	No menu management system used
29) Please identify the menu management system used at the facility	◯	◯	◯	◯	◯

30) If 'other' was selected for the last question, please provide details.

____

31) Please select all the types of menus used at the facility.

- ☐ Paper-based menu
- ☐ Digital menu
- ☐ Spoken menu
- ☐ Other

32) If 'other' was selected for the last question, please provide details.

____

33) Please indicate how digital menus are made accessible to patients at the facility.

- ☐ Mobile Apps
- ☐ Web link
- ☐ Other

34) If 'other' was selected for the last question, please provide details.

____

35) Please identify all staff members responsible for distributing menus to patients within the facility.

- ☐ Diet Aides/Nutrition Assistants/Menu Monitors
- ☐ Nurse
- ☐ Ward clerks/admin support
- ☐ Other

36) If 'other' was selected for the last question, please provide details.

____

37) Please indicate the duration of the menu cycle used within the facility.

- ◯ One-week or 7-day cycle
- ◯ Two-week or 14-daycycle
- ◯ Four week or 28-day cycle
- ◯ Six-week cycle

38) Please confirm if the facility utilises seasonal menus.

- ◯ Yes
- ◯ No

39) Please specify how frequently patients complete the menu at the facility.

- ◯ Daily
- ◯ Weekly
- ◯ Fortnightly
- ◯ Monthly
- ◯ Other

40) Please clarify whether patients receive any assistance to complete the menus with details of the type of support provided.

____

41) Please identify which of these govern menu design at the facility.

- ☐ Menu guidelines/standards
- ☐ Nutrition standards for hospital menus
- ☐ Local national dietary guidelines
- ☐ No standards or guidelines
- ☐ Other

42) If 'other' was selected for the last question, please provide details.

____

43) Please specify the individual/s responsible for ensuring that the food items at the facility adhere to the menu and nutritional guidelines.

- ☐ Foodservice dietitian
- ☐ Clinical dietitian
- ☐ Consultant dietitian (private)
- ☐ Chef
- ☐ Foodservice manager
- ☐ Medical team
- ☐ Speech Pathology team
- ☐ Nurses

44) Please specify the number of therapeutic diets available at the facility.

____

45) Please specify the number of texture-modified diets offered at the facility.

____

46) Please confirm whether the facility uses the International Dysphagia Diet Standardisation Initiative (IDDSI) framework.

- ◯ Yes
- ◯ No

47) Please indicate how the texture and consistency of the solids and fluids for texture-modified diets are assessed at the facility by selecting all that applies.

- ☐ Using objective measures (e.g., fork drip test, finger test)
- ☐ Speech Pathologists testing/approving meals and drinks
- ☐ Dietitians testing/approving meals and drinks
- ☐ Chef checks
- ☐ Other

48) If 'other' was selected for the last question, please provide details.

____

49) Please identify the methods used to evaluate food service provided to patients at the facility.

- ☐ Patient satisfaction surveys
- ☐ Plate waste audit
- ☐ Patient interviews or focus groups
- ☐ Menu assessment
- ☐ Other

50) If 'other' was selected for the last question, please provide details.

____

SECTION 4: NUTRITION PRACTICES RELATED TO FOOD SERVICES

51) Please confirm whether a clinical dietitian is involved in the facility's food services briefly outlining how.

____

52) Please identify all the tools used by dietitians in the facility to assess patients' oral intake.

- ☐ 24-hour recall (single or repeated)
- ☐ Food records
- ☐ Diet history
- ☐ Food Frequency Questionnaire
- ☐ CBORD's Mobile Intake
- ☐ Other

53) If 'other' was selected for the previous question, please provide details.

____

54) Please indicate the daily energy (kJ or kcal) target/goal of a regular menu (full or normal diet and thin fluids). Write NA if not applicable or unsure if not known.

____

55) Please indicate the daily protein (grams) target/goal of a regular menu (full or normal diet and thin fluids). Write NA if not applicable or unsure if not known.

____

56) Please indicate the daily saturated fat (grams or % energy) target/goal of a regular menu (full or normal diet and thin fluids). Write NA if not applicable or unsure if not known.

____

57) Please choose all options that confirm the facility's menu flexibility in accommodating patients' dietary requirements.

- ☐ Customised menu serves (small, regular, large sized meals)
- ☐ Customised diet code (diabetes diet, low energy diet etc)
- ☐ No customisation possible
- ☐ Other

58) If 'other' was selected for the last question, please specify.

____

59) Do dietitians at the facility evaluate diet quality as a component of the routine nutritional assessment of patients?

- ◯ Yes
- ◯ No

60) If you answered 'yes' to the previous question, please specify the names of the diet quality assessment tools used. If not applicable, simply state 'NA'.

____

61) Please indicate whether patients at the facility receive any education on making menu selections during admission.

- ☐ Yes, by dietitians
- ☐ Yes, by diet aides or nutrition assistants
- ☐ Yes, by ward administration support
- ☐ No
- ☐ Unsure

62) Please select all the food outlets from which patients obtain their meals and beverages during their stay at the facility.

- ☐ Vending machines
- ☐ Cafes/kiosks
- ☐ Local restaurants/takeaway shops
- ☐ Uber eats/other delivered services
- ☐ Family and friends

63) Please indicate whether there any guidelines or policies governing foods sourced from outside the hospital food services.

- ○ Yes
- ○ No

64) Please provide information on any additional aspects of foodservice management within the facility not addressed in this survey. If there is nothing to add, please indicate 'NA'.

____

Thanks so much for completing this survey, your contribution is greatly appreciated.

S2: Excerpt of the content validity form Excerpt

#	Question		Response		Relevance	Clarity	Text - comments
SECTION 1: YOUR DETAILS							
1	I am a dietitian currently working (or have worked previously) within spinal facilities. E.g., Acute or rehabilitation centres, private or public hospitals with dedicated spinal beds/units.	FALSE	Yes		Relevant	Clear	
		FALSE	No				
2	I have been a dietitian in the area of spinal cord injury (SCI) for	FALSE	< 1 year		Irrelevant	Clear	
		FALSE	1-5 years				
		FALSE	5-10 years				

S2a: Summary of content validity results

Summary of questionnaire sections	% Relevance	% Clarity	Comments	Actions
**Section 1**	100%	100%		
**Section 2**	100%	100%	"If other, do they need to specify what?"	Add a follow-on question to describe 'other'
Question that was flagged as lacking clarity				
The hospital/health facility is/was located in a Large Metropolitan area b Rural or remote area	100%	50%	"Why have you said 'large' if specifying size shouldn't you add small or just leave as metropolitan or rural?"	Removed 'large'
**Section 3**	100%	100%		
Question that was flagged as lacking clarity				
Describe the facility's dining model addressing both a and b below. a. Service delivery (e.g., room service, service, traditional tray service etc.) b. Dining environment (e.g., communal dining, room dining, outside garden etc)	100%	75%	"What does just 'service' mean'?"	Revised this into two separate questions under dining model.
**Section 4**	100%	100%		
Questions that were flagged as lacking clarity				
Do nutrition assessments of patients conducted by registered/accredited/clinical dietitians include comprehensive nutrient intake analysis?	100%	75%	"Not clear what this means - are you asking that a detailed analysis is undertaken e.g. food works? Also do you need to ask if it occurs for every client or only some?"	Changed this question using a dropdown list of tools
What specific nutrient targets/goals per day does the default regular menu (full or normal diet and thin fluids) meet for these nutrients below? Energy, protein, saturated fat, calcium and dietary fibre.	100%	75%	"Would be good to separate individually so that they are each clearly covered"	Created separate questions for key nutrients
Do dietitians provide routine education to patients on making menu selections during admission? Briefly outline how.	100%	75%	"Should you add other options e.g. DA, nurse, Other???"	Changed this question with a dropdown list
Are patients allowed to source food and drinks from outside during their hospital stay?	100%	75%	"The term outside is a little confusing - could this be changed to other food/drink not provided by the standard food service"	Changed this as a dropdown list question
Where do patients usually source the outside food/s from? Multiple responses can be selected.	100%	75%	"The term outside is a little confusing - could this be changed to other food/drink not provided by the standard food service"	Changed this as a dropdown list question - combined as one question with the previous row
Are there any guidelines governing foods brought from outside during patient stay at the facility?	100%	75%	No comments provided but implied lack of clarity from previous comments	Rephrased the question for clarity
Other general feedback			Ensure spelling consistency - centralized vs centralised, spell out words instead of abbreviations e.g., Food service vs FS etc.	Updated spelling for consistency. Revised phrasing where relevant based on feedback received
**Are there any specific items that you think are missing from the questionnaire?**	NA	NA	None provided	NA
**Are there any specific items that you think are unnecessary in the questionnaire?**	NA	NA	None provided	NA

Note: Data collected from n=4 experts

S3: Other Aspects of Foodservice Systems and Dining Models

Facility type	*Acute* *n=3*	*Rehabilitation* *n=10*	*Others** *n=2*
Production Systems			
Conventional/traditional		4 (27)	1 (7)
Commissary/centralized	1 (7)	1 (7)	
Ready prepared	1 (7)	1 (7)	
Combination	1 (7)	4 (27)	1 (7)
Midmeal dining model			
Same as main meals	3 (20)	5 (33)	2(13)
Different to main meals		5 (33)	
Midmeal choices			
Hot beverages	1 (7)		
Variety of midmeals[+]	2 (13)	10 (67)	2 (13)
Cutlery and Crockery			
Biodegradable	3 (20)	5 (33)	1 (7)
Non-biodegradable		1 (7)	
Combination		4 (27)	1 (7)
Mealtime support			
Feeding assistance	3 (20)	10 (67)	1 (7)
Meal set-up & cut up	3 (20)	10 (67)	1 (7)
Supervision	3 (20)	10 (67)	1 (7)
Companionship	1 (7)	5 (33)	1 (7)
Digital menu types			
Mobile apps/weblinks		3 (20)	
No digital menu	3 (20)	7 (47)	2 (13)
Menu distribution			
Food service staff or diet aides	2 (13)	7 (47)	1 (7)
Nurses or ward clerks		1 (7)	
Combination	1 (6)	2 (13)	
*Not applicable *****			1 (7)
Menu assessment			
Dietitians	2 (13)	1 (7)	
Chef/food service manager		2 (13)	
Combination	1 (7)	7 (47)	2 (13)
IDDSI			
Yes	3 (20)	8 (53)	1 (7)
No		2 (13)	1 (7)
TMD compliance measures			
Objective measures [‡‡] */speech pathologist*	1 (7)	5 (33)	1 (7)
Chef and/or dietitian	1 (7)	1 (7)	1 (7)
Combination	1 (7)	4 (27)	
Food service evaluation			
Satisfaction surveys		2 (13)	1 (7)
Menu review or plate waste		1 (7)	
Combination	2 (13)	7 (47)	1 (7)
Other [‡‡‡]	1 (7)		

*Note. Percentages are rounded and may not add up to 100. *Co-located facility (n=1); unknown setting type (n=1).* [ƒ]*4 main meals (n=1), 3x main meals and self-served snacks (n=3), combination (n=1).* [+] *combination of hot and cold beverages, bakery items, fresh or tinned fruits..* [‡‡]*fork drip test, finger test.* [‡‡‡] *Typically completed by managers within foodservice.*

S4: Summary of Free-text Comments (n=9)

**Free-text comments**
"Currently experiencing ++ issues with food service at our facility leading to patient dissatisfaction and poor PO intake which in turn is causing weight loss and malnutrition. Mainly stemming from lack of proper training provided to food service workers and quality of meals provided." (Participant 1)
"The food service management is still not so strong and needs more work out with regular monitoring and evaluation. Need of full-time dietitian and which is still lacking due to no funding in the rehabilitation centre." (Participant 2)
"We are currently working on insourcing our food service to better meet our patients individual needs + more quality control of the food provided. This will hopefully be in place by 2030." (Participant 3)
"Menus are tweaked through year but not seasonal list of diets would be larger if counted all individual allergy and intolerances and included each modified texture as one diet." (Participant 4)
"Our SCI rehab facility is soon to undergo a significant kitchen renovation which will allow us to transition to a 'cook fresh on demand' model. We are in the process of engaging with consumers to help us determine what this foodservice model looks like, what the menu will look like. The goal is that it will be a combination of what consumers want and what the research tells us is beneficial from a nutrition and environment perspective. Stay tuned :)" (Participant 5)
"OWI policy for food brought from home. Long-stay/"ward extras" list available for additional main meal choices for lunch and dinner. Some flexibility for special orders available. 'Healthy Choice' diet code available." (Participant 6)
"Dietitian constantly raised their concerns about the lack of patient satisfaction with the menu and regularly met with the food service manager to try and solve issues. Unfortunately the food service department was bound by food safety policy and cost which led to the 'solutions' being offered of little value to patients." (Participant 7)
"Meal management procedure discourages the provision/storage of externally produced cooked meals to patients." (Participant 8)
The food service at our hospital does not cater to long stay patients at all. Our patients are highly dissatisfied with the food quality, lack of variety and overall service. Regular audits display high plate wastage, poor nutritional intake and a strong dissatisfaction with the food/food service. Whilst Dietetics strongly advocate for change within these wards of exception, however the overarching food service companies show little engagement. We essentially feel powerless for our patients. (Participant 9)
